# MicroRNA-155 Promotes Heat Stress-Induced Inflammation via Targeting Liver X Receptor α in Microglia

**DOI:** 10.3389/fncel.2019.00012

**Published:** 2019-02-04

**Authors:** Ping Li, Gong Wang, Xiao-Liang Zhang, Gen-Lin He, Xue Luo, Ju Yang, Zhen Luo, Ting-Ting Shen, Xue-Sen Yang

**Affiliations:** ^1^Laboratory of Extreme Environmental Medicine, Department of Tropical Medicine, Army Medical University, Chongqing, China; ^2^Department of Neurology, Xinqiao Hospital, Army Medical University, Chongqing, China; ^3^Department of Cardiology, Kunming General Hospital of Chengdu Military Command, Yunnan, China

**Keywords:** heat stress, microRNA-155, inflammation, LXRα, microglia

## Abstract

**Background:** The neuroinflammatory responses of microglial cells play an important role in the process of brain dysfunction caused by heat stroke. MicroRNAs are reportedly involved in a complex signaling network and have been identified as neuroinflammatory regulators. In this study, we determined the biological roles of microRNA-155 in the inflammatory responses in heat-stressed microglia and explored the underlying mechanisms.

**Methods:** MicroRNA-155 mimic and inhibitor were used to separately upregulate or downregulate microRNA-155 expression. The activation state of BV-2 microglial cells (BV-2 cells) was assessed via immunoreactions using the microglial marker CD11b and CD68. Levels of induced interleukin-1β (IL-1β), interleukin-6 (IL-6) and tumor necrosis factor-α (TNF-α) were measured using real-time reverse transcription polymerase chain reaction (RT-PCR) and enzyme linked immunosorbent assays (ELISAs). The activation of nuclear factor kappa B (NF-κB) signaling proteins was evaluated by Western blotting for inhibitory kappa B alpha (IκBα) and NF-κB p65 phosphorylation and indirect immunofluorescence analysis using a p65 phosphorylation antibody. A luciferase reporter assay was used to verify liver X receptor α (LXRα) as a target gene of microRNA-155.

**Results:** Heat stress significantly induced IL-1β, IL-6, and TNF-α release and increased the expression of CD11b and CD68. In addition, IκBα and NF-κB p65 phosphorylation were dramatically increased by heat stress, and microRNA-155 expression was also elevated. High expression of microRNA-155 in heat-stressed microglial cells was inversely correlated with LXRα expression. We then determined the role of microRNA-155 in the heat stress-induced inflammatory responses. The results revealed that by targeting LXRα, microRNA-155 enhanced NF-κB signaling activation and facilitated immune inflammation in heat stress-treated BV-2 cells.

**Conclusion:** MicroRNA-155 promotes heat stress-induced inflammatory responses in microglia. The underlying mechanisms may include facilitating inflammatory factors expression by increasing NF-κB pathway activation via targeting LXRα.

## Introduction

Heat stroke is an overheated environment or high-intensity manual work- caused serious illness in which central nervous system (CNS) dysfunction predominates. Symptoms of CNS dysfunction, such as loss of consciousness, ataxia, coma, and delirium are also the main basis of diagnoses of heat stroke in the clinic (Bouchama, 2002; Mattis and Yates, 2011; Leon and Bouchama, 2015; Hifumi et al., 2018). The central pathophysiological mechanisms underlying CNS dysfunction during heatstroke are believed to involve the following aspects: direct damage to the CNS induced by hyperthermia; and the secondary insult resulting from the widespread process of cerebral ischaemia and hypoxia (Sharma and Hoopes, 2003; Yan et al., 2006; Chen et al., 2013). Although neuroinflammation is still not fully understood, increasing data have indicated that this process contributes to the consequences of CNS damage during heat stroke (Biedenkapp and Leon, 2013; Lin et al., 2018). An obvious neuroinflammatory response (NIR) has been clinically and experimentally verified (Guerrero et al., 2013; Lee et al., 2015; Chauhan et al., 2017). All these findings indicate that excessive activation of inflammation in the CNS may be the major pathological mechanism of heat stroke, and elucidation of the cellular mechanisms underlying the inflammatory response may provide guidelines for heat stroke prevention and therapy.

Microglia are the main immune cells in the CNS and can function as macrophages in the brain (Ginhoux et al., 2013). In serious neuropathological conditions, microglia can be activated and secrete proinflammatory cytokines and neurotoxic mediators, such as TNF-α, IL-6, IL-1β, NO and ROS, which cause additional neuroinflammation and aggravate brain disease progression (Biber et al., 2014; Brown and Vilalta, 2015; Dwyer and Ross, 2016; Beckers et al., 2018). Activated microglia have been found in the cerebral cortex, hippocampus and pituitary gland of the brain in heat stroke animal models. Interestingly, Biedenkapp and Leon (2013) revealed that expression of the activation markers of microglia was consistent with the profiles of cytokines and chemokines in heat stroke mice. These results indicated that microglia may play a pivotal immune modulatory role in the CNS during heat stroke. Microglia can be activated by various physical factors, such as electromagnetic radiation, ionizing radiation and infrasound (Hwang et al., 2006; Du et al., 2010; Yang et al., 2010). However, whether heat as another physical factor can directly active microglia is still unclear.

As a physical stress, high ambient temperature activates the proinflammatory response of microglia; this reaction may not occur through the classical adaptor- receptor signaling pathway, but it may change the expression levels of some mediators that indirectly influence inflammation. Recent studies have indicated that epigenetic mechanisms, such as microRNAs (miRNAs) and DNA methylation, participate in neuro-inflammation in neuropathological conditions (Thounaojam et al., 2013; Saika et al., 2017; Zhang et al., 2017; Cheray and Joseph, 2018; Wang X. et al., 2018). MiRNAs are an important class of epigenetic regulators that can regulate gene expression post-transcriptionally. A deep sequence data analysis revealed that compared to the negative control, miRNA-155, a well-studied inflammatory miRNA, increased 2.73-fold in peripheral blood mononuclear cells of heat-stressed cattle (Sengar et al., 2018), indicating the involvement of miR-155 in the heat stress-induced inflammatory response. Consistent evidence has indicated that miR-155 upregulation in neurological conditions is associated with enhancement of CNS inflammation and pathological changes (Lopez-Ramirez et al., 2014; Woodbury et al., 2015). Moreover, miR-155 was the first reported miRNA to be directly linked to microglial activation. Indirect evidence has shown that the inhibition of miR-155 ameliorates the pathogenesis of ischaemic stroke in mice by decreasing the production of proinflammatory mediators, and this process was proven to be mediated by the NF-κB signaling pathway in macrophages (Wen et al., 2015; Pena-Philippides et al., 2016). Further studies have indicated that miR-155 exerts proinflammatory effects by targeting different mediators of inflammatory signaling, including LXRα, SOCS-1, SHIP1 and transforming growth factor beta-activated kinase 1 and MAP3K7-binding protein 2 (TAB2) (Ceppi et al., 2009; Cardoso et al., 2012; Miller et al., 2013). Among these, LXRα, an oxysterol-activated transcription factor, is widely expressed in growing or mature neurons and glia and has also been shown to be a direct anti-inflammatory target of miR-155 (Miller et al., 2013; Skerrett et al., 2014; de Wit et al., 2017; Kurowska-Stolarska et al., 2017).

Considering the relationship between miR-155 and inflammatory-activated microglia, this study attempted to ascertain whether heat stress can directly activate microglia and induce a proinflammatory response *in vitro*. Is miR-155 involved in the heat stress-induced proinflammatory response and how does it influence the inflammation stimulated by heat stress? In this study, we employed a heat stressed-BV-2 cell model to answer these questions. Our preliminary data showed that miR-155 may function as a promoter in heat stress-induced inflammatory responses in BV-2 cells. The underlying mechanisms may include enhancing inflammatory factors expression by increasing NF-κB signaling pathway activation via targeting LXRα.

## Materials and Methods

### Cell Culture and Treatment

The immortalized murine BV-2 microglial cell line was purchased from the American Type Culture Collection (ATCC) and was cultured in Dulbecco’s modified Eagle’s medium (DMEM, Gibco, NY, United States) supplemented with 10% heat-inactivated fetal bovine serum (FBS, HyClone), 2 mM glutamine, 100 U/ml penicillin, 100 μg/ml streptomycin and 50 μM 2-mercaptoethanol (Sigma-Aldrich, St. Louis, MO, United States). Cells were plated in six-well flat-bottom plates at a density of 5 × 105/ml and maintained at 37°C in a humidified atmosphere of 5% CO2. Cells were passaged every 48 h with a 1:4 split ratio and used at passages 3–10. After a 24 h incubation, miR-155 mimic, miR-155 inhibitor or their respective controls (Gene Pharma Co., Ltd.) were transfected at a final concentration of 100 nM/ml in Opi-MEM (Life Technologies GmbH, Darmstadt, Germany) using Lipofectamine^®^ 2000 (Invitrogen, Life Technologies). Then, cells were treated with or without TO901317 for 1 h (10 nM, Sigma). Subsequently, cells were subjected to heat stress for 2 h in a prewarmed incubator at 42°C, followed by a recovery period at the normal growth temperature of 37°C. We collected the cell culture supernatant for enzyme linked immunosorbent assays (ELISAs) and extracted total RNA and protein for real-time PCR and Western blot analyses.

### Cell Viability Assay

Following transfection in BV-2 cells, cell viability was evaluated using the Cell Counting Kit-8 assay (CCK-8; Dojindo, Shanghai, China) according to the manufacturer’s instructions. This method is used to determine the number of metabolically active and viable cells in cell culture based on several proliferation-related elements in the cells, including dehydrogenase, NAD(H), NADP(H) and mitochondrial activity. In brief, cells were seeded in a 96-well plate at a density of 5 × 10^3^ cells/well for 24 h and transfected with synthetic miR-155 mimic, miR-155 inhibitor or their respective controls at a final concentration of 50 nM/ml using Lipofectamine^®^ 2000. After transfection for 24 h, 10 μl CCK-8 reagent was added to each well for 2.5 h. The absorbance at 450 nM was measured using a plate reader (Bio-tek Epoch, Winooski, VT, United States).

### Real-Time PCR Analysis of mRNA Levels

Total RNA was isolated using TRIzol^®^ reagent (Invitrogen, Carlsbad, CA, United States). Briefly, 800 ng of total RNA was reverse transcribed into cDNA using the PrimeScript^TM^ RT reagent Kit according to the manufacturer’s protocol. The synthesized cDNA was used as the template for the real-time PCR amplification that was carried out by the Bio-Rad CFX 96^TM^ Real-Time PCR Detection System (Bio-Rad) using a KAPA SYBR^®^ FAST qPCR kit (Kapa Biosystems, Boston, MA, United States). Specific primers were designed based on entries in the GenBank database using Primer 5.0 software (Premier Biosoft International, Palo Alto, CA, United States). Reaction conditions for real-time PCR were 3 min at 95°C, followed by 40 cycles of 3 s at 95°C and 20 s at 60°C; every cycle was followed by melt curve conditions of 65 and 95°C with increments of 0.5°C for 5 s, followed by the final plate read. HPRT served as an internal control for sample normalization, and the comparative cycle threshold method was used for data quantification. The primer sequences are shown as follows: TNF-α: Fwd 5′- GAC CCT CAC ACT CAG ATC ATC TTC T- 3′; Rev 5′-CCT CCA CTT GGT GGT TTG CT-3′. IL-6: Fwd 5′-TGG TGT GTG ACG TTC CCA TTA-3′; Rev 5′-CAG CAC GAG GCT TTT TTG TTG-3′. IL-6: Fwd 5′-ACA ACC ACG GCC TTC CCT ACT T-3′; Rev 5′-CAC GAT TTC CCA GAG AAC ATG TG-3′. iNOS: Fwd 5′-GGC AGC CTG TGA GAC CTT TG-3′; Rev 5′-GCA TTG GAA GTG AAG CGT TTC-3′. HPRT: Fwd 5′-GTT AAG CAG TAC AGC CCC AAA-3′; Rev 5′-AGG GCA TAT CCA ACA AAC TT-3′.

### Real-Time PCR Analysis of miRNAs

Total RNA, including miRNA, was extracted using TRIzol^®^ reagent (Invitrogen, Carlsbad, CA, United States). In brief, 1000 ng of total RNA was reverse transcribed into cDNA using the MicroRNA First-Strand Synthesis and MiRNA Quantitation Kits (TaKaRa, Japan) according to the manufacturer’s instructions. We performed quantitative RT-PCR analyses for miRNAs using the Mir-X^TM^ miRNA qRT-PCR SYBR^®^ Kit (TaKaRa) in a Rotorgene 6000 Thermocycler (Corbett Life Science) using the following parameters: 95°C for 3 min, followed by 40 cycles of 95°C for 15 s, 60°C for 30 s, and 70°C for 10 s. We used U6 small nuclear RNA as an endogenous control for data normalization and calculated the relative expression using the comparative threshold cycle method 2^-ΔΔCT^.

### Enzyme-Linked Immunosorbent Assays

After the designated treatment, we evaluated the secretion levels of TNF-α, IL-6 and IL-1β in cell supernatants using enzyme immunoassay kits (eBioscience, San Diego, CA, United States) according to the manufacturer’s instructions. The results of the ELISAs were read using a plate reader (Bio-tek Epoch) at 450 nm.

### Luciferase Reporter Assay

The wild type LXRα-3′UTR and mut-LXRα-3′UTR dual luciferase reporter vectors (Promega, United States) were synthetized and tested by Gene Pharma Co., Ltd., and then, BV-2 cells were cotransfected with miR-155 mimic or negative control. Cells were also transfected with the pmirGLO-control vector, which is useful for monitoring the transfection efficiency. After 24 h, the firefly luciferase activity was determined using the dual luciferase reporter assay system (Promega, United States) with a GloMAX 20/20 Luminometer (Promega, United States). We obtained the relative reporter activity through normalization to the Renilla control.

### Immunoblotting

Cells were lysed in RIPA-containing protease and phosphatase inhibitors (Roche, Penzberg, Germany). Nuclear protein was extracted by nuclear protein extraction kit (Beyotime Biotechnology, Shanghai, China) as per the company’s protocol. Protein concentration was determined using the BCA assay (Beyotime Biotechnology, Beijing, China). The solubilized denatured protein (20 ng) was subjected to electrophoresis on polyacrylamide SDS gels and transferred to polyvinylidene difluoride (PVDF) membranes (Bio-Rad, Munich, Germany). The membranes were incubated with 5% fat-free dry milk in TBST buffer at room temperature for 1 h. We then probed the membranes with primary antibodies against Glyceraldehyde 3-phosphate dehydrogenase (GAPDH) (Santa Cruz Biotechnology, Heidelberg, Germany), IκBα, pSer32/36-IκBα, NF-κB p65, pSer536-NF-κB p65 (Cell Signaling Technology, Danvers, MA, United States), LXRα (Abcam, Santa, United States) or proliferating cell nuclear antigen (PCNA) (Santa Cruz Biotechnology, Heidelberg, Germany) overnight at 4°C. After the membranes were washed with TBST, they were incubated with horseradish peroxidase-conjugated anti-rabbit or anti-mouse secondary antibody for 1 h. The immune-reactive proteins were visualized using enhanced chemiluminescence (ECL) reagent (Bio-Rad, Munich, Germany) followed by exposure to X-ray film and quantified by using Image Lab analysis software. The data were first normalized against the internal standard GAPDH and then expressed as fold changes compared to the controls.

### Immunofluorescence Staining

Cells were cultured on coverslips (10 mm × 10 mm) coated with poly-1-lysine in 24-well plates. After fixation with 4% paraformaldehyde and permeabilization with 0.3% Triton X-100, the cells were incubated in goat serum (Zhongshan Golden Bridge Biotechnology, Beijing, China) for 60 min at room temperature to block non-specific binding sites. Cells were incubated with mouse anti-mouse cluster of differentiation molecule 11b (CD11b), pSer536-NF-κB p65 or rabbit anti-mouse cluster of differentiation 68 (CD68) (1:200; Cell Signaling Technology, Danvers, MA, United States) antibody overnight at 4°C in a humidified chamber. After being washed with PBS, cells were incubated with highly cross-adsorbed CF555-conjugated donkey anti-mouse IgG secondary antibody (1:250, Sigma-Aldrich) at 37°C for 60 min in the dark. Then, the cells were counterstained with 4′ 6-diamidino-2-phenylindole dihydrochloride (DAPI; Beyotime Biotechnology, Shanghai, China). Representative fluorescence photographs were taken by an LSM 780 confocal laser scanning microscope (400× magnification, Carl Zeiss GmbH, Jena, Germany). The photographs were analyzed by ZEN 2012 light edition software (Carl Zeiss, Jena, Germany).

### Statistical Analysis

Three or more separate experiments were performed, and statistical analyses were carried out using Prism5 software (GraphPad, San Diego, CA, United States). The results are presented as the mean ± standard deviation (SD). We determined significant differences by Student’s *t*-test (two groups) or one-way ANOVA (multiple comparisons). Statistical significance was established when *p* < 0.05. ^∗^ denotes *p* < 0.05, ^∗∗^ denotes *p* < 0.01, and ^∗∗∗^ denotes *p* < 0.001.

## Results

### Heat Stress Provokes Proinflammatory Responses and Induces Microglial Activation

**Figure 1:**
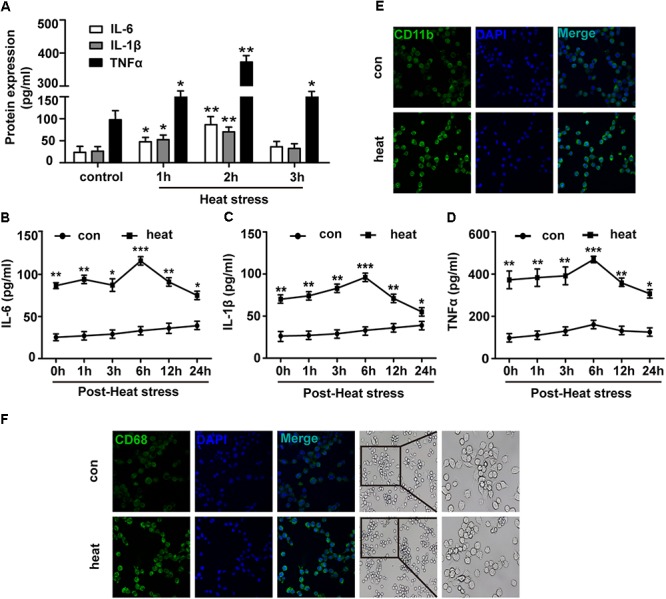
Heat stress provokes proinflammatory responses and induces microglial activation. **(A)** BV-2 cells were incubated at 37°C (control) or were subjected to heat stress treatment at 42°C for 1, 2, or 3 h. The culture medium supernatants were collected, and the protein contents of IL-6, IL-1β, and TNF-α were assayed by ELISAs. **(B–D)** Cells were subjected to a heat stress treatment at 42°C for 2 h, followed by a recovery period at 37°C for 0, 1, 3, 6, 12, or 24 h. The protein contents of IL-6, IL-1β, and TNF-α were assayed by ELISAs. **(E,F)** Cells were subjected to a heat stress treatment at 42°C for 2 h, followed by a recovery period at 37°C for 6 h. Confocal immunofluorescence microscopy was performed on cells that were immunoreacted with antibodies against CD11b and CD68 after the treatment. The images are presented at a 400× magnification. The morphology of cells was captured by inverted microscope. The images are presented at a 100 and 200× magnification. The results are presented as the mean ± SD of three independent experiments. Statistical comparisons to the control group are indicated by ^∗^*p* < 0.05, ^∗∗^*p* < 0.01, ^∗∗∗^*p* < 0.001.

To investigate the effects of heat stress on the inflammatory response of BV-2 cells, we initially examined the protein expression levels of IL-6, TNF-α and IL-1β. As presented in Figure 1A, the expression levels of IL-6, TNF-α, and IL-1β in the culture medium supernatants were differently increased following heat stress at 42°C for 1, 2, and 3 h and peaked at 2 h of exposure (*p* < 0.01). Thus 2-h heat stress was identified as a threshold condition representing the time of duration beyond which intensified alteration of growth characteristics of tested cell line occurs (data not shown). With the extension of time after 2 h of heat stress, IL-6, TNF-α, and IL-1β expression increased gradually, peaked at 6 h recovery period, and were sustained up to 24 h after heat stress, compared to that of the corresponding control group (Figure 1B–D; *p* < 0.001). Activated microglia were previously suggested to express different markers. Among these, CD11b and CD68 have the greatest biological significance (Hoogland et al., 2015; Yang et al., 2018). Because increased expression of CD11b and CD68 are a typical feature of microglial activation (Fernando et al., 2006; Roy et al., 2006), we examined the effect of heat exposure on the expression of CD11b and CD68 in BV-2 cells by confocal microscopy. Heat stress was found to significantly increase CD11b and CD68 expression compared with that of the control group and the morphology of BV-2 cells changed from ramified to amoeba in the heat stress group (Figure 1E,F). These results indicate that heat stress provoked proinflammatory responses and induced microglial activation.

### Heat Stress Could Increase miR-155 Expression in Microglia

**Figure 2:**
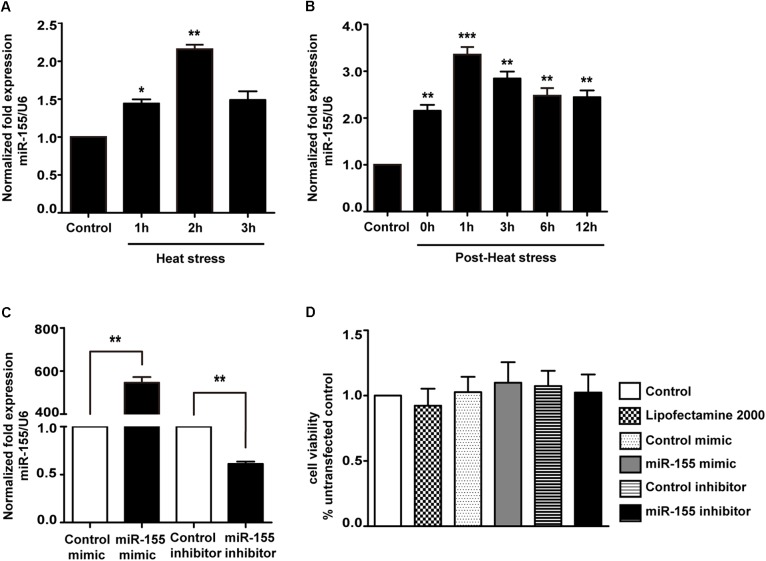
Expression of miR-155 in BV-2 cells induced by heat stress and transfection efficacy of the miR-155 mimic and inhibitor. **(A)** BV-2 cells were incubated at 37°C (control) or were subjected to heat stress treatment at 42°C for 1, 2, or 3 h. The expression levels of miR-155 in BV-2 cells were measured by qRT-PCR and normalized to U6 expression. **(B)** Cells were subjected to a heat stress treatment at 42°C for 2 h, followed by a recovery period at 37°C for 0, 1, 3, 6, 12, or 24 h. The expression levels of miR-155 in BV-2 cells were measured by qRT-PCR and normalized to U6 expression. **(C)** After 24 h of cell transfection, miR-155 expression was measured by qRT-PCR and normalized to U6. The results are presented as the fold change with respect to the control. **(D)** Cell viability was measured using CCK-8 assays after cell transfection with miR-155 mimic (50 nM), inhibitor (50 nM) or their respective controls. The untransfected control was set to 100%. Statistical analyses were performed by using a *t*-test (two groups) or one-way ANOVA with a *post hoc* Student–Newman–Keuls test (multiple comparisons). The results are expressed as the mean ± SD of three independent experiments. Statistical comparisons to the control group are indicated by ^∗^*p* < 0.05, ^∗∗^*p* < 0.01, ^∗∗∗^*p* < 0.001.

Because miR-155 is involved in peripheral inflammation and immune responses (Tili et al., 2007; Mashima, 2015), we investigated its regulation in heat stress-treated microglia. A significant increase in miR-155 expression was observed following heat stress at 42°C for 1 and 2 h (Figure 2A; *p* < 0.05). In contrast, heat stress did not affect miR-155 expression at 3 h of heat exposure (*p* > 0.05). With the extension of recovery time after 2 h of heat stress, miR-155 expression peaked at 1 h, and was sustained up to 24 h compared to the control group (Figure 2B; *p* < 0.01). Upregulation of miR-155 after heat stress suggests that miR-155 is involved in the regulation of heat stress-triggered inflammatory responses in microglia. To address this issue, we transfected BV-2 cells with miR-155 mimic, inhibitor, or their respective controls. The transfection efficacy determined by qRT-PCR showed that transfection with the miR-155 mimic increased miR-155 expression (*p* < 0.01), whereas the miR-155 inhibitors significantly decreased its availability (*p* < 0.01) compared to those of cells transfected with negative controls, indicating effective transfection (Figure 2C). To exclude the possibility that transfection could affect the survival of BV-2 cells, we performed a cell viability assay. As shown in Figure 2D, neither miR-155 mimic nor inhibitor nor their transfection reagent Lipofectamine2000 exerted any adverse effects on cellular survival. Interestingly, cells transfected with the miR-155 mimic showed an increased trend in cell viability, although no statistical significance was achieved.

### Role of miR-155 in Activating Heat Stress-Induced Inflammatory Responses in Microglia

**Figure 3:**
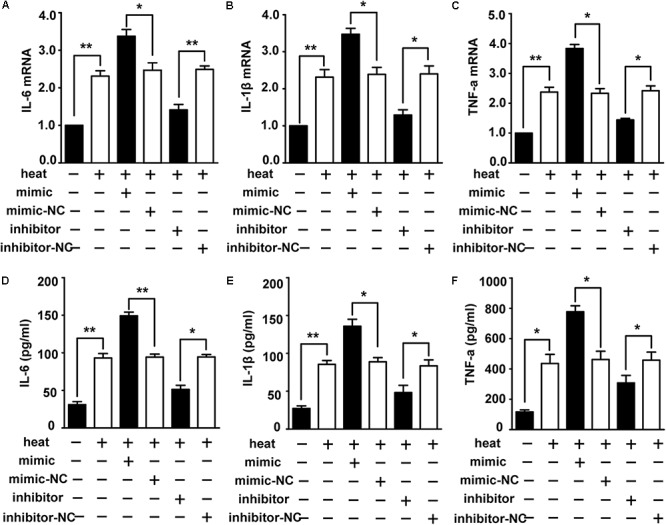
Role of miR-155 in activating heat stress-induced inflammatory response in microglial cells. **(A–F)** BV-2 cells were transfected with miR-155 mimic, inhibitor, or their respective control for 24 h. Then, the cells were subjected to a heat stress treatment at 42°C for 2 h, followed by a recovery period at 37°C for 6 h. The mRNA and protein levels of IL-6, IL-1β, and TNF-α were assessed by qRT-PCR **(A–C)** and ELISA **(D–F)**, respectively. Statistical analyses were performed by using a *t*-test (two groups). The results are expressed as the mean ± SD of three independent experiments (^∗^*p* < 0.05, ^∗∗^*p* < 0.01).

To identify the role of miR-155 in activating the heat stress-induced inflammatory response in microglia, we examined the impacts of miR-155 on the expression of IL-6, IL-1β, and TNF-α. The results (Figure 3A–F) showed that overexpression of miR-155 upregulated the mRNA and protein levels of IL-6, IL-1β and TNF-α (*p* < 0.05). In contrast, inhibition of miR-155 downregulated the mRNA and protein levels of IL-6, IL-1β and TNF-α. These data suggest that miR-155 is involved in the regulation of the proinflammatory response induced by heat stress.

### miR-155 Enhances NF-κB Activation in Heat-Stressed Microglia

**Figure 4:**
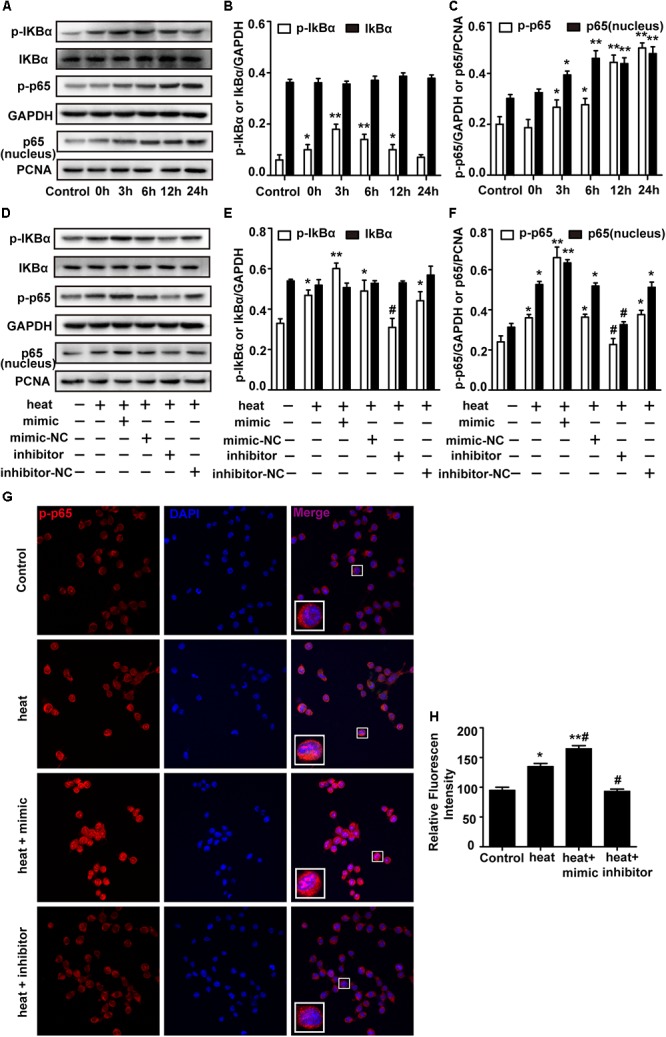
miR-155 enhances NF-κB activation in heat-stressed microglia. **(A–C)** Cells were subjected to a heat stress treatment at 42°C for 2 h, followed by a recovery period at 37°C for 0, 3, 6, 12, or 24 h. Total protein expression levels of IκBα, p-IκBα, p-p65, GAPDH and nucleus protein expression levels of p65 and PCNA were determined by Western blotting. Densitometric analysis was performed. **(D–F)** BV-2 cells were transfected with miR-155 mimic, inhibitor, or their respective controls for 24 h. Then, the cells were subjected to a heat stress treatment at 42°C for 2 h, followed by a recovery period at 37°C for 6 h. Total protein expression levels of IκBα, p-IκBα, p-p65, GAPDH and nucleus protein expression levels of p65 and PCNA were determined by Western blotting. Densitometric analysis was performed. **(G,H)** After the treatment, the cells were fixed and processed for indirect immunofluorescence analysis using the antibody against p-p65. Representative images and the analysis are shown. Statistical analyses were performed by using a *t*-test (two groups) or one-way ANOVA with a *post hoc* Student–Newman–Keuls test (multiple comparisons). The results are expressed as the mean ± SD of three independent experiments. Statistical comparisons to control group are indicated by ^∗^*p* < 0.05, ^∗∗^*p* < 0.01. Statistical comparisons to the heat stress group are indicated by #*p* < 0.05.

The NF-κB pathway is considered the central regulator of inflammatory cytokines and enzymes, such as IL-6, IL-1β, and TNF-α (Lawrence, 2009; Nguyen et al., 2014). Recent studies showed that NF-κB was activated during the recovery period following heat stress in HeLa cells and human umbilical vein endothelial cells (HUVECs) (Kretz-Remy et al., 2001; Liu et al., 2015). Therefore, we investigated whether heat stress activates NF-κB in microglia. Western blot assays were performed, and the results showed that the phosphorylation levels of IκBα and p65 and the level of p65 translocation to nucleus were low at 37°C but immediately increased after heat stress treatment. The phosphorylation was further increased during the 24 h recovery time (Figure 4A–C). However, there were no significant IκBα changes during the recovery periods, which indicates that NF-κB activation may be accompanied by phosphorylation of IκBα and p65 but not by bulk degradation of IκBα in BV-2 cells. To further study the role of miR-155 in the activation of NF-κB, we used miR-155 mimic and inhibitor in heat-stressed BV-2 cells. Western blot results showed that the miR-155 mimic significantly increased IκBα and p65 phosphorylation and the level of p65 translocation to nucleus, but the miR-155 inhibitor downregulated this phosphorylation (Figure 4D–F). The IκBα changes were also not affected by miR-155 treatment. Furthermore, indirect immunofluorescence studies demonstrated that the distribution of p-p65 in the nucleus was obviously increased after 6 h of heat stress recovery at 37°C, and miR-155 aggravated these changes, while the miR-155 inhibitor restrained the changes (Figure 4G,H). Taken together, these results suggest that heat stress induces the activation and translocation of NF-κB during the recovery period in BV-2 cells and that miR-155 enhances NF-κB activation and translocation. These findings indicated that miR-155 can regulate the inflammatory response induced by heat stress through the NF-κB signaling pathway in microglia.

### Liver X Receptor α Is a Functional Target of miR-155

**Figure 5:**
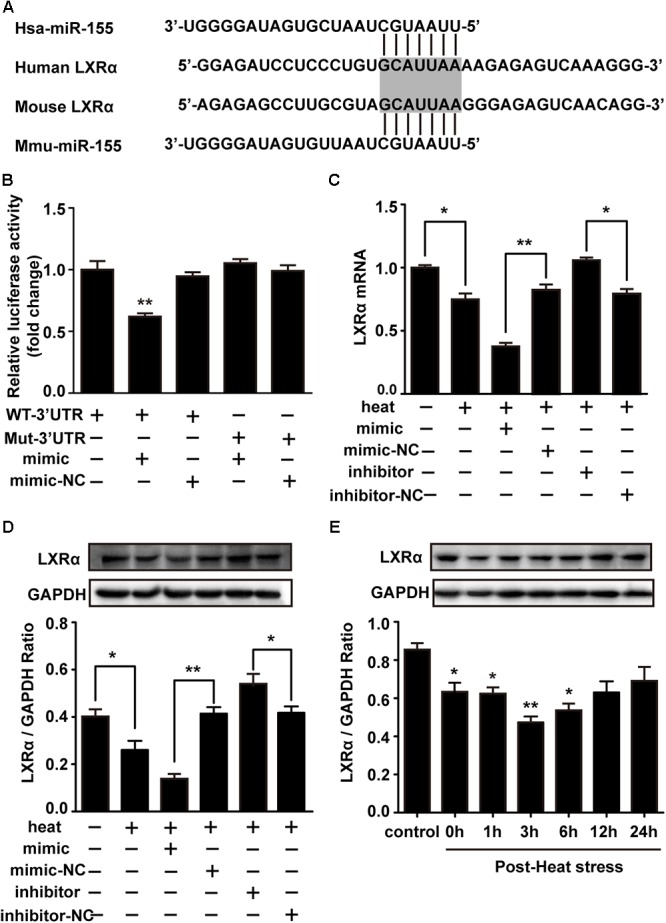
Liver X receptor α is a functional target of miR-155. **(A)** A potential target site for human and mouse miR-155 in the 3′UTR of the LXRα mRNA. The seed region is highlighted. **(B)** Luciferase reporter assays were used to validate miR-155 binding of the LXRα 3′UTR in BV-2 cells. The luciferase reporter plasmids carrying the WT or Mut 3′UTR of LXRα and miR-155 mimic or mimic-NC were cotransfected into BV-2 cells for 24 h, and then, luciferase activity was detected. ^∗^*p* < 0.01, relative to the mimic group. **(C,D)** BV-2 cells were transfected with miR-155 mimic, inhibitor, or their respective controls for 24 h. Then, the cells were subjected to a heat stress treatment at 42°C for 2 h, followed by a recovery period at 37°C for 6 h. Western blot and qRT-PCR were used to assess the protein and mRNA expression of LXRα. **(E)** Cells were subjected to a heat stress treatment at 42°C for 2 h, followed by a recovery period at 37°C for 0, 1, 3, 6, 12, or 24 h. The protein contents of LXRα were assayed by Western blot. Statistical analyses were performed by using a *t*-test (two groups) or one-way ANOVA with a *post hoc* Student–Newman-Keuls test (multiple comparisons). The results are expressed as the mean ± SD of three independent experiments. Statistical comparisons to the control group are indicated by ^∗^*p* < 0.05, ^∗∗^*p* < 0.01.

LXRα has been shown to inhibit the expression of lipopolysaccharide (LPS)-induced IL-1β, IL-6, and TNF-α and thus has a critical role in the regulation of inflammation (Wang J. et al., 2018). Two different online database searchers, TargetScan^1^ and miRanda^2^, predicted that the 3′UTR of the LXRα mRNA transcript in mice contains putative binding sites for miR-155, and the binding site appears to be highly conserved in humans (Figure 5A). Next, we performed luciferase reporter assays in the cells to determine whether LXRα is a direct target of miR-155. Cotransfection of agomir-155 with the luciferase reporter gene linked to the wild-type (WT) segment of the LXRα 3′UTR strongly repressed luciferase activity (*p* < 0.05), and the luciferase activity of the mutant was significantly rescued (Figure 5B). These findings demonstrate that miR-155 can directly target the 3′UTR of LXRα mRNA through this binding site. To identify whether the miR-155-LXRα pathway is active in heat-exposed BV-2 cells, we separately transfected cells with miR-155 mimic, miR-155 inhibitor or their respective negative controls. As shown in Figure 5C,D, qPCR and Western blot analyses indicated that overexpression of miR-155 inhibited LXRα expression at both the mRNA and protein levels, while inhibition of miR-155 resulted in a significant increase in LXRα mRNA and protein levels (*p* < 0.05) in heat-stressed BV-2 cells. Thus, miR-155 can directly target LXRα in BV-2 cells. Next, we examined the role of LXRα in BV-2 cells during various periods of recovery time. Western blotting showed that LXRα expression was rapidly inhibited and was maintained at low levels for more than 6 h before increasing to baseline levels after a 12 h recovery period (Figure 5E). These results indicated the threshold 6 h recovery time of heat stress with prominent proinflammatory activity.

### Exogenous LXRα Agonist Ameliorates the Effects of miR-155 and Heat Stress on the Inflammatory Responses and NF-κB Activation in Microglia

**Figure 6:**
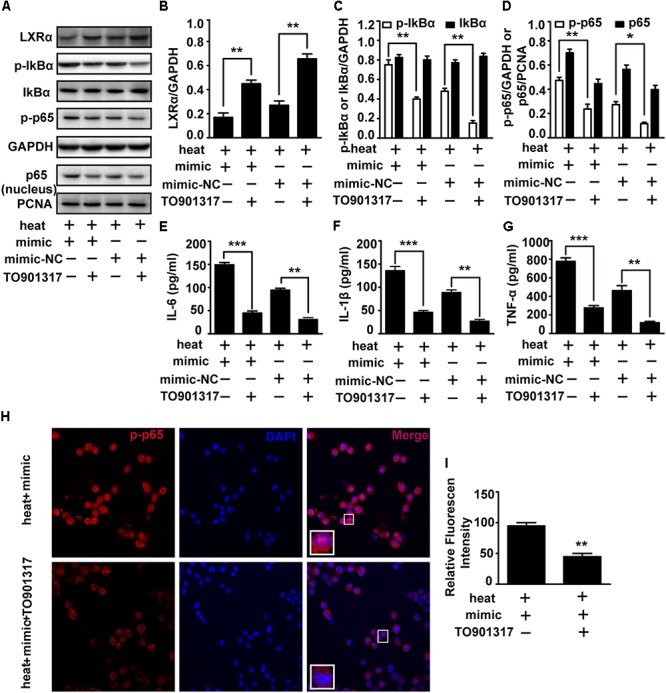
An exogenous LXRα agonist ameliorates the effects of miR-155 and heat stress on the inflammatory responses and NF-κB activation in microglia. BV-2 cells were pretreated with or without 10 nM/ml TO901317 1 h before being transfected with miR-155 mimic or mimic control. Then, the cells were subjected to a heat stress treatment at 42°C for 2 h, followed by a recovery period at 37°C for 6 h. **(A)** Total protein expression levels of LXRα, IκBα, p-IκBα, p-p65, GAPDH and nucleus protein expression levels of p65 and PCNA were determined by Western blotting. **(B–D)** Densitometric analysis was performed. **(E–G)** The protein expression levels of IL-6, IL-1β, and TNF-α were assessed by ELISAs. **(H,I)** Indirect immunofluorescence analysis using the antibody against p-p65. Representative images and the analysis are shown. Statistical analyses were performed by using a *t*-test. The results are expressed as the mean ± SD of three independent experiments (^∗^*p* < 0.05, ^∗∗^*p* < 0.01, ^∗∗∗^*p* < 0.001).

To test whether LXRα is involved in mediating the effects of miR-155 on inflammatory responses and NF-κB activation, we used the LXRα agonist TO901317 to elevate the LXRα expression downregulated by miR-155 mimic and heat stress. The addition of TO901317 significantly reversed the decreased LXRα expression induced by miR-155 mimic and heat stress, indicating effective treatment of LXRα agonist (Figure 6A,B). Meanwhile, the elevated phosphorylation of IκBα and p65 and the level of p65 translocation to nucleus induced by the miR-155 mimic and heat stress was also reversed by TO901317 (Figure 6A,C,D). Furthermore, indirect immunofluorescence studies demonstrated that the distribution changes of p65 in the nucleus were reversed by TO901317 (Figure 6H,I). Moreover, TO901317 blocked the upregulated protein levels of IL-6, IL-1β, and TNF-α induced by miR-155 and heat stress (Figure 6E–G). Taken together, these observations suggest that the effects of miR-155 on inflammatory responses and NF-κB activation are dependent on the LXRα pathway.

## Discussion

The inflammatory response plays a critical role in the injury process following hyperthermia in the CNS. In the present study, we observed a significant increase in miR-155 and proinflammatory cytokines after 2 h of heat stress at 42°C in BV-2 microglial cells. Moreover, inhibition of miR-155 significantly reduced the levels of proinflammatory cytokines, such as IL-6, IL-1β, and TNF-α. Overexpression of miR-155 augmented the release of these cytokines. These results indicated that miR-155 is involved in the heat stress-induced inflammatory response in BV-2 cells. Mechanistically, the NF-κB signaling pathway was found to be activated in heat-stressed BV-2 cells, and miR-155 overexpression could promote the NF-κB signaling pathway by inhibiting LXRα translation. In contrast, this enhanced inflammatory response could be alleviated by a specific agonist of LXRα, TO901317. Our results demonstrated that miR-155 overexpression controls LXRα activity, with subsequent NF-κB activation, thereby causing a robust promotion of the proinflammatory response in heat-stressed BV-2 cells.

Microglia are the inherent immune effector cells of the CNS and play a significant role in the inflammatory response of the CNS. Many stimuli, such as surface structures of bacteria, abnormal endogenous proteins, cytokines and physical stress, have been reported to trigger the transformation of resting microglia to activated states (Hwang et al., 2006; Du et al., 2010; Yang et al., 2010; Radler et al., 2014; Jin et al., 2016; Hoogland et al., 2018). Recently, accumulating evidence has demonstrated that heat stress as a physical stimulus can induce microglial activation in the brain (Biedenkapp and Leon, 2013; Kim et al., 2015; Lee et al., 2015). However, whether such activation is induced directly by heat stress or results from central nervous injury is still unknown. In this study, we observed a dramatic increase in CD11b expression in an *in vitro* model by exposing BV-2 cells to heat stress conditions. Our results confirmed that heat stress could directly induce microglial activation *in vitro*. Several studies have indicated that following activation, microglia can produce a range of inflammatory mediators, including cytokines, chemokines, prostaglandins, and NO (Herber et al., 2006; Fismen et al., 2012; Kim et al., 2018), which subsequently aggravate neuronal injury (Block et al., 2007). In addition, a number of studies showed that the expression of cytokines, such as IL-6, IL-1β, and TNF-α, was elevated in the CNS of heat-exposed mice (Biedenkapp and Leon, 2013; Lee et al., 2015; King et al., 2017). Consistently, our results showed significantly increased release of the proinflammatory factors IL-6, IL-1β, and TNF-α in BV-2 cells after heat exposure. These results suggest that heat stress, as an external physical factor, could facilitate microglial proinflammatory responses through the secretion of proinflammatory factors. Thus, this microglial reactivity may ultimately contribute to brain inflammation and related neurotoxicity under heat stress conditions.

The inflammatory response is characterized by coordinated activation of inflammatory mediators and various signaling pathways. NF-κB has long been considered an essential transcription factor for the induction of inflammatory mediators, such as COX-2, IL-6, IL-1β and TNF-α (Lawrence, 2009). When NF-κB is associated with inhibitory molecules of the IκB family in the cytosol, it is inactive. Correspondingly, the activation of NF-κB involves IκB phosphorylation and the translocation of the NF-κB dimer from the cytoplasm to the nucleus (Lawrence, 2009). Here, our experiments showed that heat stress immediately increased the phosphorylation levels of IκBα and p65; moreover, the phosphorylation was further increased during the recovery time. However, the expression of IκBα was not significantly changed in this study. These results indicate that NF-κB pathway activation induced by heat stress may be accompanied by phosphorylation of IκBα and p65 but not by bulk degradation of IκBα in microglia. Furthermore, other studies demonstrated that NF-κB signaling was activated by heat stress and contributed to the inflammatory responses in macrophages or apoptosis in HUVECs (Kretz-Remy et al., 2001; Lee et al., 2015; Liu et al., 2015; Hop et al., 2018). These observations suggest that heat exposure likely affects microglial activation and cytokine release through the activation of the NF-κB pathway.

Inflammation-related gene expression in the brain can be regulated not only by transcription factors at the transcriptional level but also by miRNAs at the post-transcriptional level. Many miRNAs play an important role in heat-related disease (Roufayel et al., 2014; Liu et al., 2017). Given that miR-155 is upregulated by heat stress in peripheral blood mononuclear cells, as shown by deep sequence data analysis (Sengar et al., 2018), and participates in the proinflammatory responses (Faraoni et al., 2009), we hypothesized that miR-155 was deeply involved in regulating heat stress-induced microglial activation and inflammation. The present results showed that the expression of miR-155 was elevated following heat exposure. In addition, ectopic high levels of miR-155 could increase, and blocking miR-155 could decrease, the production of IL-6, IL-1β, and TNF-α induced by heat stress. Meanwhile, miR-155 enhanced NF-κB activation in heat-stressed microglia. These results were consistent with those of other reports, which showed that miR-155 enhanced NF-κB signaling and cytokine release in oxidized low density lipoprotein (oxLDL)-stimulated macrophages (Yang et al., 2015; Ye et al., 2016). These results indicated that miR-155 acts as a promoter of inflammatory responses in heat-stressed microglia via the NF-κB pathway.

Among the miR-155 targets, LXRα attracted our attention. LXRs are oxysterol-activated nuclear receptors that play a pivotal role in cholesterol homeostasis, glycolipid metabolism and the inflammatory response (Zelcer, 2006; Yoon et al., 2013). In the CNS, LXRs are widespread in growing or mature neurons and glial cells (Mandrekar-Colucci and Landreth, 2011). Recent studies showed that LXRα could disrupt NF-κB activation by a process called *trans*-repression and thus has an anti-inflammatory function (Zhang-Gandhi and Drew, 2007; Mandrekar-Colucci and Landreth, 2011; Cui et al., 2012; Canavan et al., 2013). Therefore, we investigated the role of miR-155-LXRα in regulating the heat stress-induced inflammatory response to elucidate the underlying mechanism. First, we observed a significant inverse correlation between miR-155 and LXRα expression after heat exposure in microglia. Using a luciferase reporter assay, we verified LXRα as a target of miR-155 in heat-induced microglia. To test whether LXRα is involved in mediating the effects of miR-155 on inflammatory responses and NF-κB activation, we used the LXRα agonist TO901317 (Wu et al., 2016; Paterniti et al., 2017) to elevate the LXRα expression downregulated by the miR-155 mimic and heat stress. The results showed that the elevated activation of NF-κB by the miR-155 mimic and heat stress was reversed by TO901317. Moreover, TO901317 blocked the upregulated protein levels of IL-6, IL-1β, and TNF-α induced by miR-155 and heat stress. Taken together, these observations suggest that the effects of miR-155 on inflammatory responses and NF-κB activation are dependent on the LXRα pathway. Furthermore, we found that TO901317 only partially reversed the changes evoked by the miR-155 mimic, suggesting that other targets might be involved in miR-155’s function in heat stress-induced microglia.

Chronic inflammation has long been hypothesized to be a driving force in various diseases. Exploring the mechanism of inflammation in different cell types can provide support to the treatment of inflammation-related diseases. It has been demonstrated klotho can suppress inflammation by inactivating NF-κB activation in cardiomyocytes, which make it to be a potential therapeutic agent to treat diabetic cardiomyopathy (Guo et al., 2018). Wei et al. (2016) and Zou et al. (2017) proved that memantine and Cystain C can promote the cell survival of 6 -hydroxydopamine (6-OHDA)-lesioned PC12 cells by regulating Nurr77 or VEGF, indicating a new approach for the treatment of Parkinson’s disease (PD). MPP^+^-induced inflammatory activation of BV-2 microglia may be mediated by TLR4/NF-κB inflammatory signaling, involving the further discovery of PD pathophysiology (Zhou et al., 2016). In the present study, we validated that miR-155 aggravated the inflammatory response following heat stress in BV-2 cells. Given the different patterns of inflammatory responses in various diseases, the discovery of miR-155-NF-κB pathway in heat stressed-microglia may be a new breakthrough in the treatment of CNS inflammation in heat stroke. Although we preliminary revealed this inflammatory mechanism in BV-2 cells, serial verified researches toward this issue should be concerned, especially the employment of primary microglia cultures and animal experiments.

## Conclusion

In this study, we investigated the effect of miR-155 on the heat stress-induced inflammatory response in BV-2 microglial cells. We found that excessive heat stress is a physical stimulus that can activate microglia and provoke inflammatory cytokines release. In this process, miR-155, as a regulator, enhanced inflammatory factors expression by increasing NF-κB signaling pathway activation via targeting LXRα. Regardless of the detailed mechanism, the data presented in this study may provide new insight into the mechanism of heat-related diseases.

## Author Contributions

X-SY, PL, and GW designed the research. PL, GW, X-LZ, XL, JY, ZL, and T-TS performed the experiments. PL, GW, X-SY, X-LZ, and G-LH analyzed the data. PL and X-SY wrote the manuscript. All authors reviewed the manuscript.

## Conflict of Interest Statement

The authors declare that the research was conducted in the absence of any commercial or financial relationships that could be construed as a potential conflict of interest.

## Footnotes

1 http://www.targetscan.org/

2 http://www.microrna.org/microrna/home.do

## Abbreviations

6-OHDA: 6-hydroxydopamine
COX-2: cyclooxygenase-2
IκBα: inhibitory kappa B α
IL-1β: interleukin-1β
IL-6: interleukin-6
LXRα: liver X receptor α
MPP^+^: 1-methy1-4-phenylpyridinium
NF-κB: nuclear factor kappa B
NO: nitric oxide
Nur77: nerve growth factor IB
Nurr1: nuclear receptor related 1
ROS: reactive oxygen species
SHIP1: Src homology 2-containing-inositol-phosphate-1
SOCS-1: suppressor of cytokine signaling-1
TAB2: transforming growth factor beta-activated kinase 1 and MAP3K7-binding protein 2
TLR4: Toll-like receptor 4
TNF-α: tumor necrosis factor-α
VEGF: vascular endothelial growth factor.
